# Healthcare providers and patients: an essay on the importance of professional assertiveness in healthcare today

**DOI:** 10.1080/10872981.2023.2200586

**Published:** 2023-04-11

**Authors:** Claude Richard, Marie-Thérèse Lussier, Bernard Millette, Issam Tanoubi

**Affiliations:** a Centre de Santé et des Services Sociaux de Laval, Montréal, Québec; b Département de médecine de famille et de médecine d’urgence, Université de Montréal, Québec, Canada; c Medical Simulation Centre, Centre d’Apprentissage des Attitudes et Habiletés Cliniques (CAAHC), Université de Montréal

**Keywords:** Professional assertiveness, communication, healthcare provider, patient, Healthcare system

## Abstract

Professional assertiveness can enable the healthcare provider to confidently share their expertise without seeming authoritarian to the patient. Professional assertiveness is an interpersonal communication skill that helps express opinions or knowledge while respecting similar competencies in others. For healthcare providers, this compares to sharing scientific or professional knowledge with their patients while respecting their person, ideas, and autonomy. Professional assertiveness also connects the patient’s beliefs and values with actual scientific evidence and healthcare system constraints. The definition of professional assertiveness might be easy to understand, but it remains challenging to apply in clinical practice. In this essay, we hypothesize that the practical difficulties healthcare providers encounter with assertive communication stem from their misunderstanding of this style.

## Introduction

Nowadays, information is increasingly accessible on a variety of platforms and from a multitude of sources. Some sources provide the public with rigorous and verified information, while others can produce misleading or questionable facts on important topics, such as healthcare. These sources can range from journal articles to newsprint or even social media. During the COVID-19 pandemic, the amount of information produced on the virus, vaccines, treatments, and protective measures, for example, grew exponentially. Zielinski called this proliferation of health information an ‘infodemic’ [1], or the overproduction of accurate and inaccurate information during an epidemic. He noted that it was increasingly becoming ‘difficult, if not impossible, to separate the important from the mundane, the original from the repetition, and – most worryingly – the true from the false’ [1–3].

This infodemic directly impacted, and still influences, the relationship between healthcare providers (HCP) and their patients. While the general population holds their own beliefs and attitudes towards health care and scientific information, this sheer amount of new information greatly affects how patients see, trust, or feel about healthcare and science. This shift has given HCP some nuanced challenges going forward, particularly in communicating with patients on important and life-saving topics.

People now have easier access to scientific opinions, evidence, and studies. Non-scientific professionals, such as the general public, may take these studies and their results at face value without criticism, despite these studies not going through rigorous peer review. These studies can be methodologically questionable, and their results can be erroneous. Still, patients could be profoundly influenced and believe its contents as the information is packaged as ‘scientific’ literature [3].

Rigorous, insightful, and life-saving scientific material for the general public abound, these materials can improve people’s understanding of healthcare-related issues and contexts. For patients, they can improve their understanding of treatment and therapy and help them make informed decisions about their therapeutic choices. Indeed, the greater availability of health information could also improve patient literacy in healthcare and science. By enhancing a patient’s medical knowledge, we could also improve the shared decision-making process and enhance the patient-partner approach, which is proven to be quite effective [4,5]. Ultimately, a more accurate understanding and factual information can help patients commit to their own care.

## Contextualizing the significance of professional assertiveness

The abundance of competing scientific ‘truths’ has contributed to the waning confidence in actual scientific fact. We are witnessing a post-factual era where the ‘truth’ has become closer to faith than science. At its core, science does not offer ‘truths’ but seeks to identify associations or probable casualties and prove these links with rigorous testing and data. Therefore, the advancement of technologies and the evolution of knowledge can constantly challenge accepted scientific evidence, as perhaps it should. However, the overabundance of information created by an infodemic can have two significant consequences for non-scientifically trained individuals (i.e., patients, general population) seeking healthcare information [6].

First, the value of evidence-based information can be ‘downgraded’ to the status of an opinion where one opinion is as valuable as any other. Since the same amount of data may support either and the accuracy of the data is unquestioned, the two opinions can be argued as equally ‘correct’ (i.e., *everyone is entitled to their own opinion*). The danger lies in whether one of those ‘opinions’ can directly cause public harm or erode established fact-based public health protocols. Second, the internet strongly drives alternate auto-confirming ‘health realities,’ often entirely dissociated from scientific evidence. Algorithms in search engines and social media can contribute significantly to the cognitive confirmation bias of false narratives and erroneous scientific discourse [7].

Therefore, the general public risks trusting statements they perceive as truths based on unsubstantiated evidence rather than scientific information that has undergone rigorous verification [8]. In this new context, and in a healthcare setting, simply providing information to a patient about their disease or therapy may prove insufficient to establish or maintain trust between them and their HPC [9]. In the COVID-19 pandemic and infodemic context, HCP have witnessed a shift in how they may now need to communicate with patients on health-related issues. Now, HPC may have to work extra hard to prove to or convince some populations that science is indeed *fact*, and once communicating informally in a neutral discourse about science may now require a more persuasive or professionally assertive speech.

## Defining professional assertiveness

Professional assertiveness (PA) requires both interpersonal skills and professional expertise to effectively express opinions or knowledge while simultaneously respecting those same competencies in others. In a healthcare context, PA helps the healthcare professional respectfully share important scientific knowledge without patronizing the patient and while respecting their autonomy. Demonstrating PA in healthcare must not be misconstrued as a call to dominate or force the patient. Patients assign some disciplinary authority to their HPC because they recognize their expertise and competencies. Patients gain access to these skills by consulting their HPC to identify and treat their health problems. While patients assign a sort of authority to the HCP and gain access to their skills through, they, however, should be viewed as autonomous decision-makers. By explicitly asking for their expert opinion, patients do concede ‘limited’ disciplinary authority to HCP. Still, the nature of the professional’s authority is epistemic and in no way refers to a form of control over the patient [10]. Patients can also withdraw their trust from HCP and grant it to other entities according to their expectations [11].

Patients would not respond well to a treatment plan that compelled them to subjugate themselves to a healthcare professional or institution. The patient may not prefer a pre-determined therapeutic plan. Instead, they may favour a convincing and reassuring therapeutic approach where they see themselves as a key player, a project manager, or someone slightly in charge of the way forward. So, the patient expects the physician to consult, provide understandable explanations, and enter into a mutual tacit agreement. The absence of one of these steps directly threatens the patient’s confidence in their HCP. Professionally assertive communication links patient values and evidence-based decisions through the connection with the HCP; therefore, PA is integral to any informed decision-making process [12–17]. However, while the definition of PA seems intuitive and easy to understand, it remains challenging to apply in practice.

## What does professionalism entail?

Professionalism is a structuring concept that fosters a set of similar behaviours among the members of the same profession, such as healthcare professionals. Professionalism allows those in the same field to commit to similar and coordinated behaviours to ensure patient safety and satisfaction. Patients can often identify expected professional behaviours in HCP and foster complementary behaviours [18]. For example, mental health professionals treat their patients as a primary decision-maker. They use suggestions or assertions to collaboratively integrate patient preferences and decisions into the proposed therapeutic plan. According to a study of 52 primary-care consultations for depression, anxiety, and stress, these professionals seldom used direct or authoritative statements or propositions, suggesting that they have mainly developed verbal strategies consistent with PA [19]. To be relevant to the patient, PA cannot be dissociated from professional knowledge, commitment, credibility, or reputation [20]. Doctors strongly endorse their treatment recommendations through verbal, paralinguistic, and non-verbal cues for patients to understand their therapeutic options. However, weak or poorly conveyed endorsements may negatively affect the patient’s confidence in the proposed treatment.

Some physicians adopt a cautious or conciliatory verbal style, avoiding an authoritarian approach with a sincere desire to avoid confrontation or offend their patients. This cautiousness may cause the patient to perceive the HCP as disengaged from their professional responsibilities or unsure of their knowledge, including treatment options. When the HCP attempts to avoid authoritarianism through a ‘soft’ approach, he may prevent patients from grasping the severity of the situation or the importance of the treatment plan. Consequently, this attitude toward communicating important scientific knowledge and expertise may ultimately harm the patient.

## An assertive attitude: flexible in form and firm in ideas

**Figure 1.**
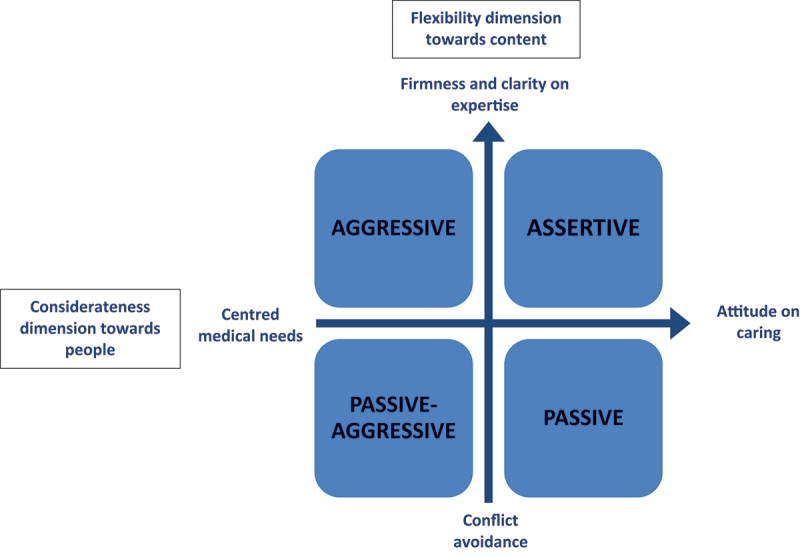
Two-dimensional professional assertiveness concept.

There is a relationship between assertiveness, self-confidence, and personality. We have adapted this relationship in the context of HCP assertiveness [21–25] in a diagram of the assertive interview that goes beyond the verbal exchange (Figure 1). We based this two-dimensional PA concept on the content of idea exchanges and the relational approach to the patient. This approach distinguishes four professional profiles: the assertive, aggressive, passive, and passive-aggressive professional.

The *assertive* professional combines firmness and clarity of language regarding their professional knowledge and skills while maintaining an attitude of benevolence and respect for others. They allow and encourage patient participation, express scientific or medical information clearly, and pay attention to the relational quality and the patient’s reaction. The *aggressive* professional is firm in expressing their knowledge and skills but imposes their views without considering those of others. Aggressive professionals do not seek collaborative relationships. The *passive* professional is flexible on ideas, always complacent, and avoids affirming their professional knowledge and skills to appear less confrontational or circumvent contention. They choose to preserve the relationship at the expense of their professional identity or beliefs. The *passive-aggressive* professional is flexible on ideas while remaining confrontational.

The assertive professional profile seems well suited to healthcare since medical and scientific knowledge expresses itself in probabilities and not certainties; however, confidence in these certainties is paramount. Patient trust is built by having confidence that the HCP knows what they are talking about (logos), is committed to that knowledge (pathos), and possesses the necessary professional credibility (ethos) conveyed by their actions and expertise [20]. Trust between the patient and HCP may weaken if these aspects are compromised. For example, patients may question the HCP’s competence, feel that they are not committed to their professional knowledge, or do not seem emotionally engaged.

## Medical encounters as polyphonic dialogue

Healthcare professionals have several ways of communicating with patients and colleagues. The HCP verbatim can be represented in a fascinating ethical framework for healthcare communication and relationships called the ‘ethics triangle’ [26]. This framework describes the actors involved in any clinical decision and organizes them with an associated pronoun. In this ‘triangle’, there are three elements: 1) the HCP’s knowledge, professional experience, and values are represented in the first person as (*I*); 2) the patient, with their own beliefs and understandings of their health, in the second-person of (*you*); 3) and finally, the context outside the HCP and the patient relationship, such as colleagues, institutions, scientific knowledge, guidelines, and the healthcare system, is represented in the third-person (*they*). When communicating with patients, the HCP often uses external examples to contextualize the situation [20,26].

In addition to these three actors, the HCP–patient relationship generates a (*we*). This ‘*we’* is how HCP and patients are referred to as a team. Using *we* associates and affirms a common approach, project, and goal [27,28]. The presence of the *we* could thus constitute an important driver of patient participation.

PA is inextricably linked to the professional *I*. The *I* amalgamates a representation of the personal *I* and the *they* (professional ethics, organizations, etc.). Words associated with the professional *I* and the *they* are often dictated to answer *we* questions (*who, what, where, when*, and *how*).

Here are a few verbatim illustrations of the polyphonic dialogue in the medical encounter.

Invocation of the *they* refers to scientific/ethical/practice guide sources: ‘It’s my job to talk to you about your sleeping pills.’

Use of the professional *I* that refers to the healthcare provider’s knowledge, recommendation, and experience: ‘*I* am confident that this new prescription will control all your symptoms.’

Use of the *you* that refers to the HCP’s patient: ‘I see *you* don’t take much. *You* only take it when needed.’

Use of the *we* that refers to a common project, an agreed plan, and the patient’s relationship: ‘Do you agree that *we* stop these pills? *We* have been talking about it for a long time.’

On the one hand, references to the *I* and *they* are relatively stable over time – these references evolve slowly. On the other hand, the *you* and the *we* are more dynamic and vary with different interlocutors. Therefore, we have qualified these references as temporary or passing.

**Figure 2.**
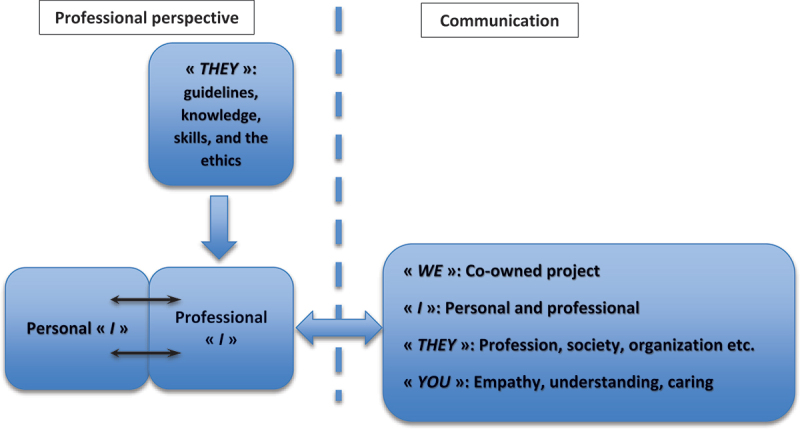
Professional behavioral mouldability.

From a normative point of view, but also because of the development of their relationship, a unique *we* can emerge. The *we* has numerous shared building blocks: shared knowledge, collaboration on specific areas, common goals, mutual respect, and so on. The *we* is associative and may take on as many expressions as possible relationships as a product of the dialogue (Figure 2). Establishing the *we* is signalling a partnership with the patient, representing the best guarantee of the long-term success of the therapeutic plan, especially for chronic diseases.

We witness a polyphonic dialogue where each (who?) responds to the other with multiple ‘voices.’ To the ‘voices’ of the HCP, the patient will respond with their own ‘voices.’ Each uses the combination of ‘voices’ they consider most appropriate to move the dialogue toward a favourable conclusion. Language, in its use, is dynamic, complex, carries multiple realities, and serves primarily to act on the other [29,30]. The HCP should consider assertiveness in a dynamic context that takes on several forms or colours depending on the evolution of the dialogue.

## Conclusion

As a communication device among healthcare professionals, professional assertiveness should not be mistaken as an attempt to force a patient to accept the directives but rather a collaborative effort with the patient to bring about a positive outcome in their healthcare experiences. Professional assertiveness is a valuable professional competence, especially in the context of the Covid-19 pandemic and infodemic, where multitudes of unverified, misleading, and even false information are now omnipresent. Advocating and affirming the value of professionally assertive discourse is critical to stemming facetious sources of information. In other words, the HCP may need to defend what the patient usually takes for granted – scientific truths. Indeed, professional assertiveness requires openness and benevolence toward the patient. Still, precise, kind, and respectful communication allows healthcare professionals to better situate the patient’s experience according to their condition and help them toward a healthier future.

## References

[1] Zielinski C. Infodemics and infodemiology: a short history, a long future. Pan American J Public Health. 2021;45:1–6. 10.26633/RPSP.2021.40 PMC8110882 33995517

[2] Zarocostas J. How to fight an infodemic. Lancet. 2020;395(10225):676. 32113495 10.1016/S0140-6736(20)30461-X PMC7133615

[3] Mheidly N, Fares J. Leveraging media and health communication strategies to overcome the COVID-19 infodemic. J Public Health Policy. 2020;41(4):410–420. 32826935 10.1057/s41271-020-00247-w PMC7441141

[4] Bourmaud A, de Villars EP, Renault-Teissier E. [Patient partnership and patient education in oncology]. Bull Cancer. 2022;109(5):588–597. 34782118 10.1016/j.bulcan.2021.09.016

[5] Haaser T, Constantinidès Y, Dejean C, et al. Démocratie sanitaire : le patient partenaire de sa prise en charge. Cancer/Radiothérapie. 2020;24(6–7):736–743. DOI:10.1016/j.canrad.2020.06.021 32861610

[6] Gisondi MA, Barber , R, Faust, JS, et al. A deadly infodemic: social media and the power of COVID-19 misinformation. J Med Internet Res. 2022;24(2):e35552. DOI:10.2196/35552 35007204 PMC8812140

[7] Lim AJ, Tan E, Lim T. Infodemic: the effect of death-related thoughts on news-sharing. Cogn Res Princ Implic. 2021;6(1):39. 34018066 10.1186/s41235-021-00306-0 PMC8136755

[8] Macagno F. Argumentation profiles: a tool for analyzing argumentative strategies. Informal Logic. 2022;42(1). DOI:10.22329/il.v42i1.7215

[9] Hargraves I, LeBlanc, A, Nilay, DS, Victor, MM, et al. Shared decision making: the need for patient-clinician conversation. Not Just Information: Health Aff (Millwood). 2016;35(4):627–629. DOI:10.1377/hlthaff.2015.1354 27044962

[10] Pilnick A, Dingwall R. On the remarkable persistence of asymmetry in doctor/patient interaction: a critical review. Soc Sci Med. 2011;72(8):1374–1382. 21454003 10.1016/j.socscimed.2011.02.033

[11] Barone S, Lazzaro-Salazar M. ‘Forty bucks is forty bucks’: an analysis of a medical Doctor’s professional identity. Lang Commun. 2015;43:27–34.

[12] Doverspike WF *Assertiveness: A key to good communication*. 2009 cited January 19]. Available from: http://drwilliamdoverspike.com/

[13] Janzen W, Myers DV. Assertion for therapists: a professional bill of rights. Psychotherapy. 1981;18:291–298.

[14] McIntyre TJ, Jeffrey, DB, McIntyre, SL. Assertion training: the effectiveness of a comprehensive cognitive--behavioral treatment package with professional nurses. Behav Res Ther. 1984;22(2): 311–8. 6466281 10.1016/0005-7967(84)90011-1

[15] Pfafman T Zeigler-Hill, Shackelford. *Assertiveness*, in encyclopedia of personality and individual differences. 2017.

[16] Richard C, Lussier M. Assertivité, professionnalisme et communication en santé (première partie). Exercer. 2019;155:322–327.

[17] Richard C, Lussier M. Assertivité, professionnalisme et communication en santé (deuxième partie). Exercer. 2019;156:369–374.

[18] Harari YN. Sapiens : une brève histoire de l’humanité. France: Albin Michel; 2015.

[19] Ford J, Thomas, F, Byng, R, McCabe, R et al. Exploring how patients respond to GP recommendations for mental health treatment: an analysis of communication in primary care consultations. BJGP Open. 2019;3:4. doi:10.3399/bjgpopen19X101670. PMC6995855 31662317

[20] Amossy R. La présentation de soi. Ethos et identité verbale (Paris : PUF). France: Argumentation & analyse du discours [En ligne]; 2010. p. 235.

[21] Berger J *L ’assertivité*. cited 2022 January 19]. Available from: https://tripole.pagesperso-orange.fr/siteFR/chroniques/assertivite_global.pdf

[22] Aucoin R. Estime de soi et assertion chez une population de couples. Québec, Canada: Université du Québec à Trois-Riviéres; 1983.

[23] Alberti RE, Emmons ML. Your perfect right: a guide to assertive behavior. 2d ed. ed. San Luis Obispo, Ca: Impact; 1974.

[24] Speed B, Goldstein B, Goldfried M. Assertiveness Training: a Forgotten Evidence-BasedTreatment. Clin Psychol: Sci Pract. 2017;25. DOI:10.1111/cpsp.12216

[25] Fensterheim H, Baer J. Don’t say yes when you want to say no: making life right when it feels all wrong. USA: Dell; Reissue edition; 1975.

[26] Boulianne S, Laurin S, Firket P. Aborder l’éthique en supervision clinique: Une approche en 3 temps. Can Fam Physician. 2013;59(7):795–797. PMC3710065 23851562

[27] Stivers T, Barnes RK. Treatment recommendation actions, contingencies, and responses: an introduction. Health Commun. 2018;33(11):1331–1334. 28825505 10.1080/10410236.2017.1350914

[28] Stivers T, Heritage, J, Barnes, KR et al. Treatment Recommendations as Actions: Health Commun. Health Commun. 2018;33(11):1335–1344. DOI:10.1080/10410236.2017.1350913. 28816510

[29] Richard C, Lussier M. *Une approche dialogique de la consultation*, in *La communication professionnelle en santé*. LMe Richard C, Editor. Québec, Canada: Pearson- ERPI; 2016. pp. 19–42.

[30] Richard C, Lussier MT, editors. *L’approche CIM : convaincre, implanter, maintenir*, in *La communication professionnelle en santé*. Québec, Canada: Pearson. 2016; pp. 233–267.

